# X-irradiated umbilical cord blood cells retain their regenerative effect in experimental stroke

**DOI:** 10.1038/s41598-024-57328-z

**Published:** 2024-03-22

**Authors:** Kazuta Yasui, Yuko Ogawa, Orie Saino, Rie Akamatsu, Akihiro Fuchizaki, Yoriko Irie, Makoto Nabetani, Mitsunobu Tanaka, Yoshihiro Takihara, Akihiko Taguchi, Takafumi Kimura

**Affiliations:** 1 Japanese Red Cross Kinki Block Blood Center, Ibaraki city, Osaka Japan; 2 Department of Regenerative Medicine Research, Foundation for Biomedical Research and Innovation at Kobe, Kobe city, Hyogo Japan; 3 Department of Pediatrics, Yodogawa Christian Hospital, Osaka, Japan

**Keywords:** Stem cells, Neurology, Cardiovascular diseases

## Abstract

Although regenerative therapy with stem cells is believed to be affected by their proliferation and differentiation potential, there is insufficient evidence regarding the molecular and cellular mechanisms underlying this regenerative effect. We recently found that gap junction-mediated cell–cell transfer of small metabolites occurred very rapidly after stem cell treatment in a mouse model of experimental stroke. This study aimed to investigate whether the tissue repair ability of umbilical cord blood cells is affected by X-irradiation at 15 Gy or more, which suppresses their proliferative ability. In this study, X-irradiated mononuclear (XR) cells were prepared from umbilical cord blood. Even though hematopoietic stem/progenitor cell activity was diminished in the XR cells, the regenerative activity was surprisingly conserved and promoted recovery from experimental stroke in mice. Thus, our study provides evidence regarding the possible therapeutic mechanism by which damaged cerebrovascular endothelial cells or perivascular astrocytes may be rescued by low-molecular-weight metabolites supplied by injected XR cells in 10 min as energy sources, resulting in improved blood flow and neurogenesis in the infarction area. Thus, XR cells may exert their tissue repair capabilities by triggering neo-neuro-angiogenesis, rather than via cell-autonomous effects.

## Introduction

Cell-based regenerative therapy is a new medical treatment that has recently evolved at a rapid pace. Among the various human cell sources, umbilical cord blood (UCB) has been getting special attention due to its several advantages. Hematopoietic or mesenchymal stem/progenitor cells present in the UCB have been shown to have superior proliferation capabilities both in vitro and in vivo compared to those in the bone marrow (BM) or adult peripheral blood (PB)^1–4^. In addition, it has been clinically proven that UCB cells are less prone to react to allogeneic immunogens than other hematopoietic cell sources^1,3^. Moreover, UCB is easy to collect with extremely low risk for the donors, and can be supplied in a short period of time as a cryopreserved product with sufficient quality control. It has also been demonstrated clinically as well as experimentally that UCB has a potent ability to evoke the tissue repair process in cerebrovascular disorders caused by hypoxic or ischemic tissue injury^5^. The intravenous administration of UCB-derived CD34^+^ cells in animals with an experimental stroke was reported to have a significant effect on the restoration of physical and cognitive capabilities, reflecting a marked recovery of the brain volume at the site of injury^6^.

Nevertheless, it remains unclear how the infused UCB-derived CD34^+^ cells exert their tissue regeneration effects. We recently demonstrated that intravenously administered allogeneic mouse BM mononuclear cells (MNC) reached the infarct site in the brain of mice with an experimental stroke to transfer low-molecular-weight substances to the endothelial cells via gap junction in just 10 min^7^. This finding raised the question of whether it is essential for the administered stem and progenitor cells to retain their hematopoietic activity in addition to their tissue regeneration ability. Therefore, we aimed to investigate whether the tissue repair ability of UCB cells is affected by X-irradiation at 15 Gy or more to ensure the immunological safety of blood products, thus depriving them of their proliferative ability. The results of this study will not only help in unravelling the precise mechanism underlying the tissue repair ability of stem progenitor cells (SPC) in UCB, but also lead to a fascinating paradigm shift in future regenerative therapies, in which cells can be transplanted for their neo-neuro-angiogenic therapeutic effects without the associated risk of graft-versus-host disease (GVHD) and further tumorigenicity.

## Results

### UCB-derived MNC enhance endothelial VEGF uptake in vitro

Recently, we established a flow cytometric method in which significantly enhanced vascular endothelial growth factor (VEGF) uptake by human umbilical vein endothelial cells (HUVEC) can be detected when cocultured with BM-MNC, with the aim of semi-quantitatively assessing the ability of hematopoietic cells to promote angiogenesis^7^.

**Figure 1:**
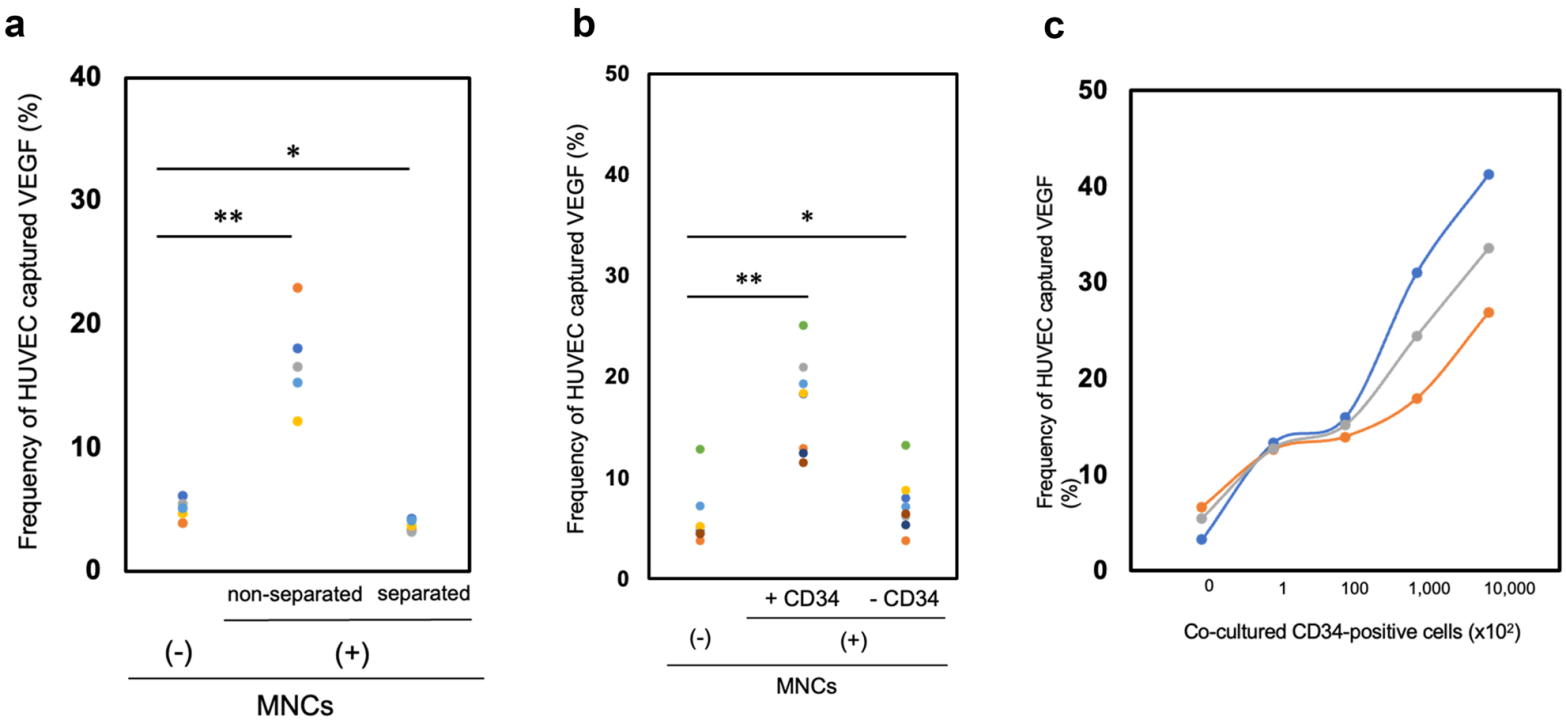
Umbilical cord blood (UCB)-derived CD34^+^ cells promote angiogenesis in vitro. (**a**) Vascular endothelial growth factor (VEGF) uptake by human umbilical vein endothelial cells (HUVEC) without coculture with UCB-derived mononuclear cells (MNC) is shown. A significant increase in the VEGF uptake by monolayered HUVEC cocultured with UCB-derived-MNC was observed, with very little uptake when MNC were cultured separately in semipermeable chambers (n = 5). (**b**) When the levels of VEGF uptake by HUVEC cocultured with CD34^+^ cells or CD34^+^ cell-depleted MNC were measured, a significant increase in the allophycocyanin (APC) fluorescence was observed in HUVEC cocultured with CD34^+^ cells but not with CD34^+^ cell-depleted MNC (n = 7). (**c**) VEGF uptake by HUVEC gradually increased in a CD34^+^ cell dose-dependent manner (n = 3). Differences were considered statistically significant at *p* < 0.05. The symbols * and ** denote *p* < 0.05 and *p* < 0.01, respectively.

We assumed that the uptake of VEGF by HUVEC may be enhanced by coculturing with UCB cells similar to that with BM-MNC. As expected, we observed a significant increase in the VEGF uptake by HUVEC when cocultured with UCB-derived MNC, while very little uptake was found when MNC were separately cultured in semipermeable chambers (Fig. 1a, Supplementary Data 1-1 (a)). These findings indicate that direct cell–cell interaction with UCB-derived MNC may be essential for the significant uptake of VEGF by HUVEC. In addition, a remarkable increase in the VEGF uptake was observed in HUVEC cocultured with MNC including CD34^+^ cells, but not in those cocultured with CD34^+^-depleted MNC (Fig. 1b, Supplementary Data 1-1 (b)). Moreover, the VEGF uptake by HUVEC gradually increased in a CD34^+^ cell dose-dependent manner (Fig. 1c, Supplementary Data 1-1 (c)). These results suggest that human vascular endothelial cells may be induced to proliferate through direct cell–cell contact with UCB-derived MNC, especially with CD34^+^ cells.

### Endothelial VEGF uptake is enhanced by MNC derived from X-irradiated UCB

**Figure 2:**
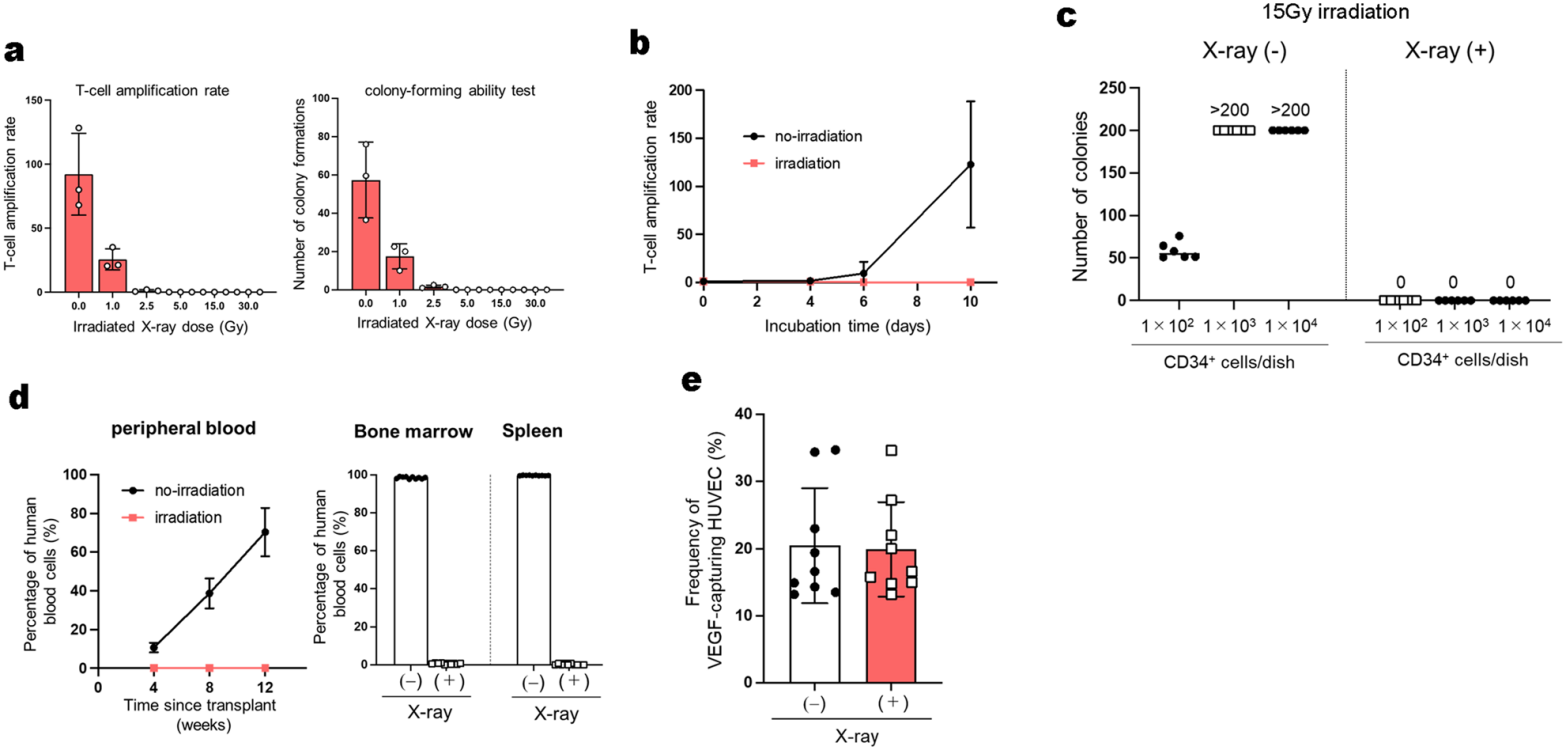
Characteristics of X-irradiated umbilical cord blood (UCB)-derived mononuclear cells (MNC). (**a**) T-cells or CD34^+^ cells were purified from UCB, which was irradiated with 0, 1, 2.5, 5, 15, or 30 Gy of X-rays, the optimal dose of which was pre-determined. Proliferative capacity of the cells was assessed by T lymphocyte activation and colony-forming unit (CFU) assays (n = 3). (**b**) Irradiation with 15 Gy of X-rays completely inhibits the proliferation of UCB-derived T lymphocytes (n = 6). (**c**) CFU-assays were used to evaluate the short-term hematopoietic ability of hematopoietic progenitor cells in UCB-derived MNC. Hematopoietic colony formation was compared between pre-irradiated and irradiated UCB-derived MNC (n = 6). (**d**) Ten of the 20 NOD/Shi-scid/IL-2RγKOJic (NOG) mice received 5.0 × 10^4^ MNC and the remaining 10 received 1.0 × 10^6^ XR cells via the tail vein. The percentage of human CD45^+^ cells was measured by flow cytometry (FCM) 4, 8 and 12 weeks after transplantation for human cells in peripheral blood and 12 weeks after transplantation for human cells in bone marrow and spleen. (**e**) Either pre- or post-irradiated UCB-MNC and HUVEC were co-cultured and the amount of VEGF incorporated into HUVEC was compared (n = 12). Differences were considered statistically significant at *p* < 0.05. All results are expressed as the mean ± standard deviation (SD).

As described in our recent report^7^, it takes only 10 min for the intravenously administrated BM-derived MNC to transfer substances to the damaged endothelium via gap junction, followed by angiogenesis in the damaged brain area. This led us to hypothesize that the proliferation and differentiation capabilities may be not essential for this rapid behavior of hematopoietic cells. We then attempted to evaluate the effect of X-irradiation on the proliferation abilities of UCB-derived MNC. X-ray irradiation at 15 Gy or more completely suppressed the short-term proliferation abilities of both T-lymphocytes and hematopoietic colony-forming cells (Fig. 2a–c, Supplementary Data 1-2 (a)–(c)). In addition, the long-term reconstitution potential of human hematopoiesis in immune-deficient mice was also completely abrogated (Fig. 2d, Supplementary Data 1-2 (d)). To examine how depriving UCB-derived MNC of their hematopoietic activity affects the VEGF uptake by HUVEC, we isolated MNC from UCB irradiated with X-ray at 15 Gy or more for coculturing with HUVEC. Notably, these X-irradiated MNC (XR cells) significantly enhanced the VEGF uptake by HUVEC to the same extent as the pre-irradiated ones (Fig. 2e, Supplementary Data 1-2 (e)). This finding led us to the conclusion that UCB-derived MNC have an inherent ability to promote angiogenesis independently of their lympho-hematopoietic activities. We observed no significant change in the percentage of apoptotic cells in UCB-derived MNC including CD34^+^ cells, T cells, B cells, and monocyte fractions for at least 48 h after X-irradiation. The X-irradiation, however, induced rapid increases in the number of apoptotic CD34^+^ or T cells only when proliferation stimuli were added to UCB-MNC (Supplementary Figure S1, Supplementary Data 4). These results indicate that X-irradiation alone did not affect the cellular composition in UCB-derived MNC within at least 48 h.

### XR cells retain their regenerative effect on an experimental stroke mouse model

**Figure 3:**
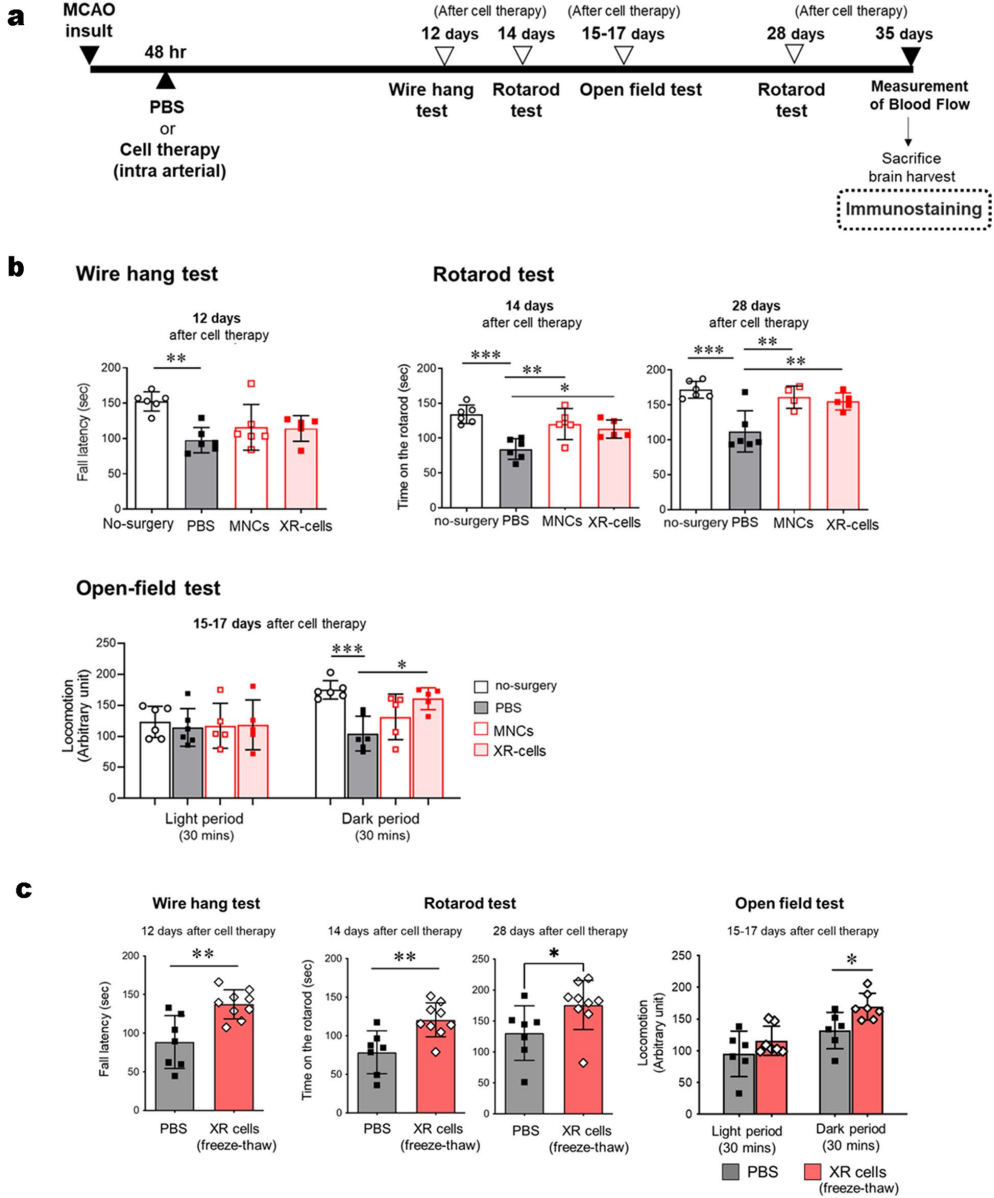
Effects of X-irradiated mononuclear cells (XR-MNCs) on a mouse model of stroke. (**a**) The experimental design is shown. (**b**) To assess motor function, mice were subjected to behavioural tests in the wire-roping test, rotarod test and open field test after MCAO procedure. All behavioural tests were performed at the optimal time based on a preliminary study. (**c**) Behavioural tests were performed with freeze-thawed XR-MNC. Differences were considered statistically significant at *p* < 0.05. All results are expressed as the mean ± standard deviation (SD). The symbols *, **, and *** denote *p* < 0.05, *p* < 0.01, and *p* < 0.001, respectively.

As previously demonstrated^6^, CD34^+^ UCB cells have potent neuro-angiogenic effects in a mouse model of experimental stroke. However, it has not been precisely elucidated how these cells contribute to the improvement of brain function. Therefore, we administrated XR cells or their parental pre-irradiated ones to mice with an experimental stroke. Behavioral evaluation was performed according to the schedule shown in Fig. 3a. It was found that the XR cell-treated group showed significant therapeutic effects in all behavioral tests, which were comparable to those in the mice treated with parental pre-irradiated MNC (Fig. 3b, Supplementary Data 3-1, 2, 3). Interestingly, the effects of XR cells were not significantly attenuated even after freeze–thaw operations (Fig. 3c, Supplementary Data 3-4, 5, 6), which might be supported by the fact that angiogenesis was predominantly promoted around the infarction area in the brain of the recipient mice.

### Metabolic changes and blood flow improvement in the brain triggered by rapid cell–cell metabolite transfer

**Figure 4:**
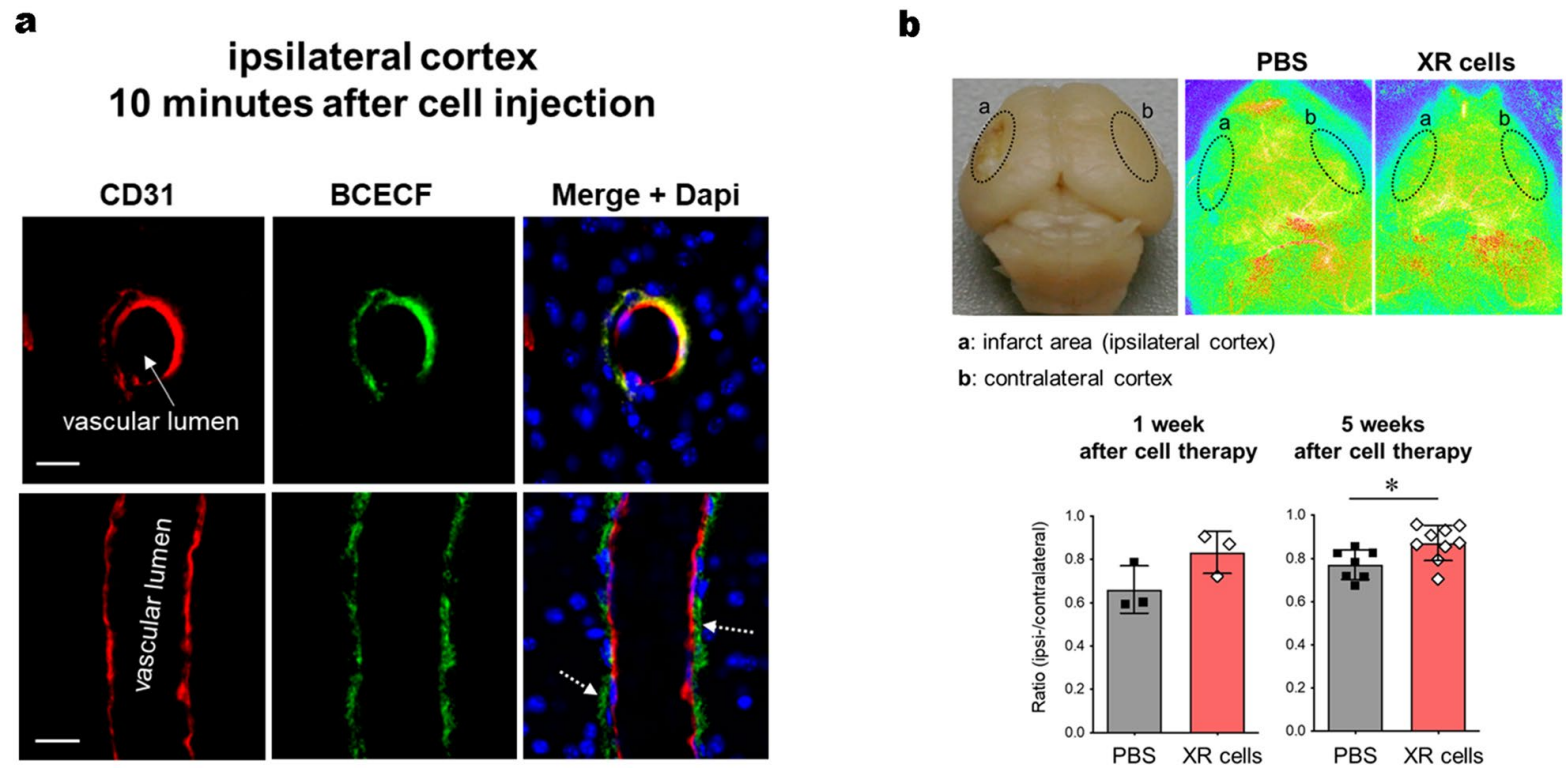
Delivery of small molecules from X-irradiated mononuclear cells (XR) to vascular endothelial cells at the injured side. (**a**) The BCECF-signals in the cerebral vascular endothelial cells from the ipsilateral cortex were evaluated using fluorescence microscopy. (Upper row) Transferred BCECF-positive signals (green) observed in the endothelial cells 10 min after XR cell transplantation. (Lower row) Transferred BCECF are also visible in non-endothelial cells located outside the vasculature. Scale bars = 10 µm. (**b**) Cerebral surface blood flow was measured in the mouse model of stroke using a laser speckle flowmetry imaging system (1 week: MCAO-PBS treatment, n = 3 and MCAO-XR cell treatment, n = 3; 5 weeks: MCAO-PBS, n = 7 and MCAO-XR cell treatment, n = 9). Representative images are shown in the top row, and their quantitative results are shown in the bottom row. Differences were considered statistically significant at *p* < 0.05. All results are expressed as the mean ± standard deviation (SD). The symbols * denote *p* < 0.05.

Next, we investigated the therapeutic mechanism of the XR cells. As described in our previous report^7^, after freeze-thawing, 2′,7′-bis-[2-carboxyethyl]-5-[6]-carboxyfluorescein (BCECF)-loaded XR cells were injected into mice 48 h after middle cerebral artery occlusion (MCAO) insult. We observed transfer of the BCECF signal from the XR cell cytoplasm to the vascular endothelial and non-endothelial cells located outside the vasculature in the ipsilateral cortex 10 min after the cell injection (Fig. 4a, Supplementary Data 3-7 and 8). This indicates that XR cells, similar to pre-irradiated MNC, can provide low-molecular-weight metabolites to the damaged cerebrovascular endothelial cells in an extremely short time of 10 min. Therefore, the therapeutic mechanism of MNC is unlikely to be altered by X-irradiation.

**Figure 5:**
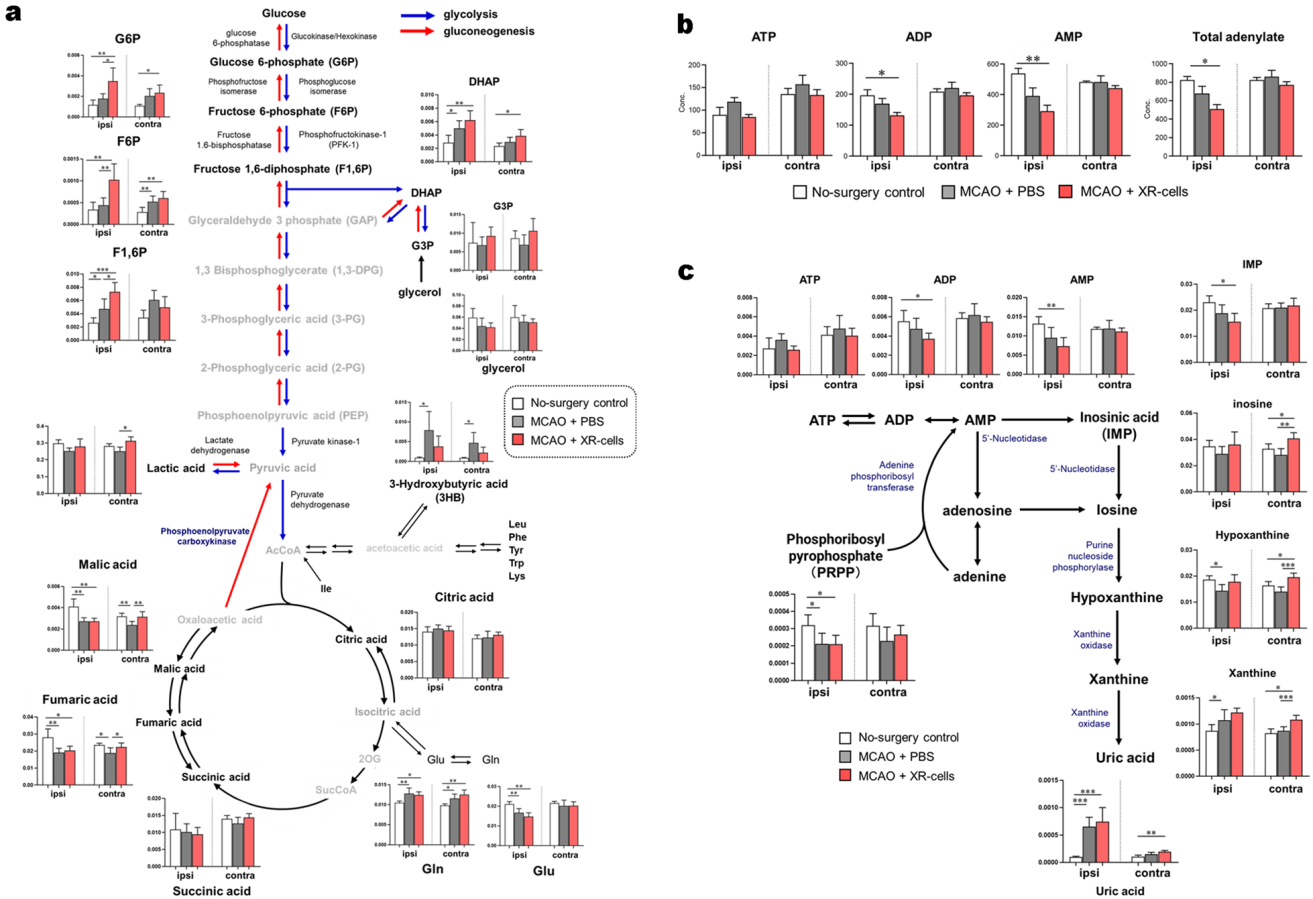
Metabolites 24 h after X-irradiated mononuclear cells (XR) therapy in a middle cerebral artery occlusion (MCAO) mouse brain model-1. (**a**) Map of the central metabolic pathway and comparative visualization of the metabolites involved in XR cell therapy. (**b**) Total adenylate and adenylate energy change. Abbreviations: ATP, adenosine triphosphate; ADP, adenosine diphosphate; AMP, adenosine monophosphate. (**c**) de novo synthesis of purine nucleotides after cell therapy. Differences in the levels of metabolites among the no-surgery control, MCAO-phosphate-buffered saline (PBS), and MCAO-XR cell groups are presented as bar graphs. ‘Ipsi’ represents the ipsilateral side, and ‘contra’ represents the contralateral side of the mouse brain. Black font indicates substances that were detected, and gray font indicates substances that were not detected in all groups. Differences were considered statistically significant at *p* < 0.05. All results are expressed as the mean ± standard deviation (SD). The symbols *, **, and *** denote *p* < 0.05, *p* < 0.01, and *p* < 0.001, respectively.

**Figure 6:**
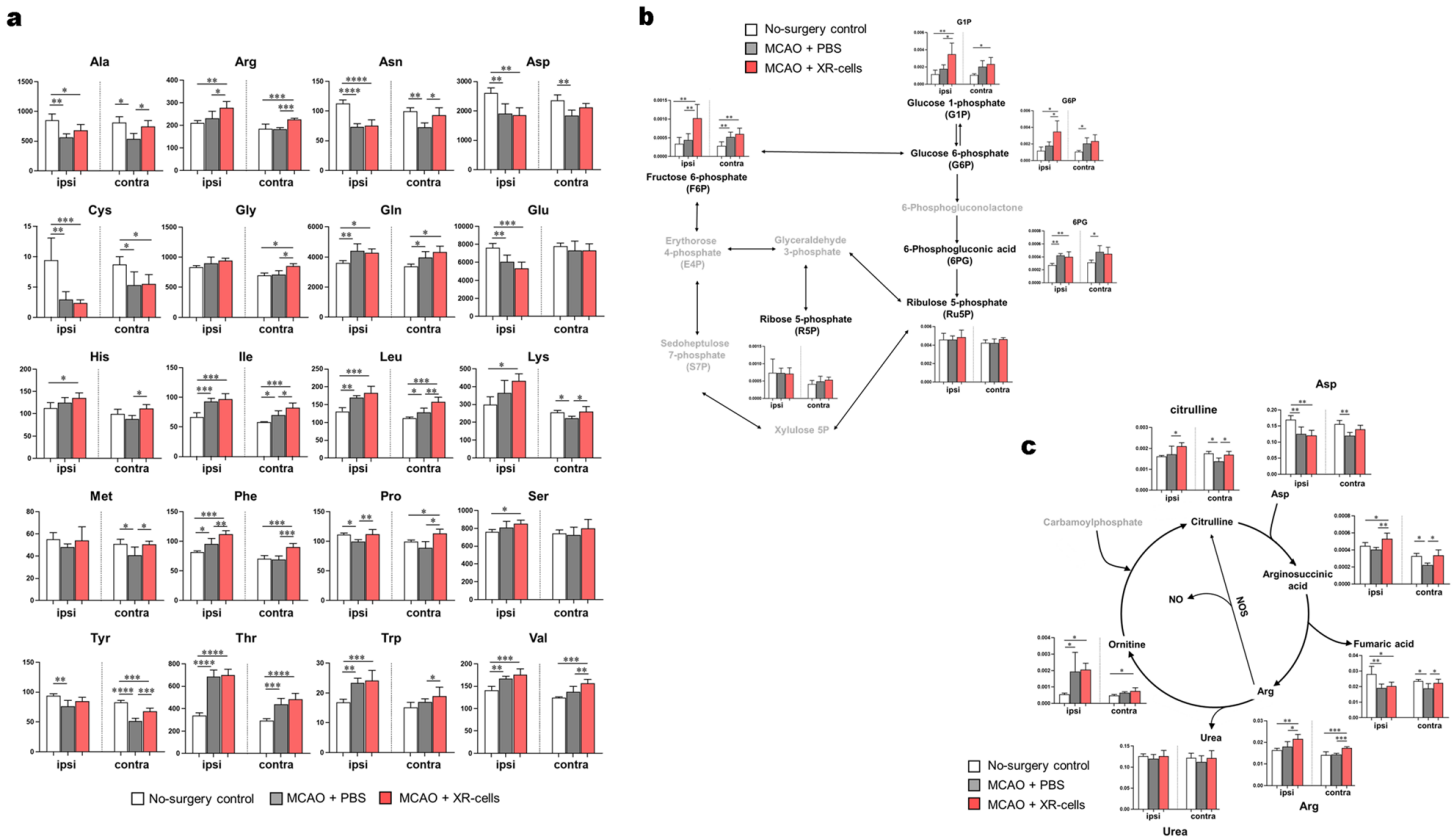
Metabolites at 24 h after X-irradiated mononuclear cells (XR) therapy in a middle cerebral artery occlusion (MCAO) mouse brain model-2. (**a**) Amino acid concentration after cell therapy. Differences in the levels of metabolites among the no-surgery control, MCAO-phosphate-buffered saline (PBS), and MCAO-XR cell groups are presented as bar graphs. ‘Ipsi’ represents the ipsilateral side, and ‘contra’ represents the contralateral side of the mouse brain. Abbreviations: Ala, alanine; Arg, arginine; Asn, asparagine; Asp, aspartic acid; Cys, cysteine; Gln, glutamine; Glu, glutamic acid; His, histidine; Ile, isoleucine; Leu, leucine; Lys, lysine; Phe, phenylalanine; Pro, proline; Ser, serine; Thr, threonine; Trp, tryptophan; Tyr, tyrosine; and Val, valine. (**b**) Pentose phosphate pathway metabolites after cell therapy (n = 5). (**c**) Urea cycle metabolites after cell therapy. Black font indicates the detected substances, and gray font indicates substances that were not detected in all groups (n = 5). Differences were considered statistically significant at *p* < 0.05. All results are expressed as the mean ± standard deviation (SD). The symbols *, **, and *** denote *p* < 0.05, *p* < 0.01, and *p* < 0.001, respectively.

We then attempted to evaluate how the initial low-metabolite transfer led to changes within 24 h in the metabolic state of the brain. Compared with the phosphate-buffered saline (PBS)-treated group, the XR cell-treated group showed significantly higher levels of glycolysis 24 h after the treatment (Fig. 5a, Supplementary Data 2). On the other hand, the XR cell-treated group had lower total adenylate levels than the PBS-treated group (Fig. 5b, Supplementary Data 2). The degradation and synthesis pathways of adenylates were then investigated (Fig. 5c, Supplementary Data 2). In the degradation pathway, the degradation product uric acid was significantly higher in the untreated and XR cell-treated groups. In terms of the synthetic pathways, PRPP and IMP, which are the starting points for purine base synthesis, showed low values in both groups. We observed significant differences in the amount of several amino acids, such as Arg, Leu, Phe, and Pro, as energy sources, between the untreated and cellular treatment groups on the injury side (Fig. 6a, Supplementary Data 2). In addition, the energy metabolite 3-HB (ketone bodies) was increased in the untreated group, while early glycolytic intermediates such as G6P and F6P were increased in the cell-treated group (Fig. 6b, Supplementary Data 2). We observed that ornithine, involved uric acid metabolism, was increased in mice after experimentally induced stroke (Fig. 6c, Supplementary Data 2). Hematopoietic stem/progenitor cells (HSPC) have been shown to remain in a high-energy state with an activated glycolytic system^8^. Presumably, the transfer of XR cell (especially CD34^+^ cell)-derived metabolites to the damaged endothelial cells results in the activation of energy metabolism in the brain of the recipient mice.

Concurrent with the metabolic activation, we found remarkably improved blood-flow in the infarction area of the recipient mice who received an infusion of XR cells (Fig. 4b, Supplementary Data 3-9 and 10). Specifically, angiogenesis was observed after 1 week in the XR cell-treated group, whereas these were not observed in the PBS-treated group. Furthermore, a comparison of cerebral surface blood flow between the XR cell group and the PBS group showed no significant difference at 1 week post-infusion, while a significant increase was observed in the XR cell group at 5 weeks post-infusion. Based on the knowledge of salient angiogenesis, blood-flow improvement in the infarction area might be influenced by precedent revascularization elicited by UCB-derived MNCs^7,9,10^.

### XR cells possibly trigger neurogenesis by penetrating the blood–brain barrier

**Figure 7:**
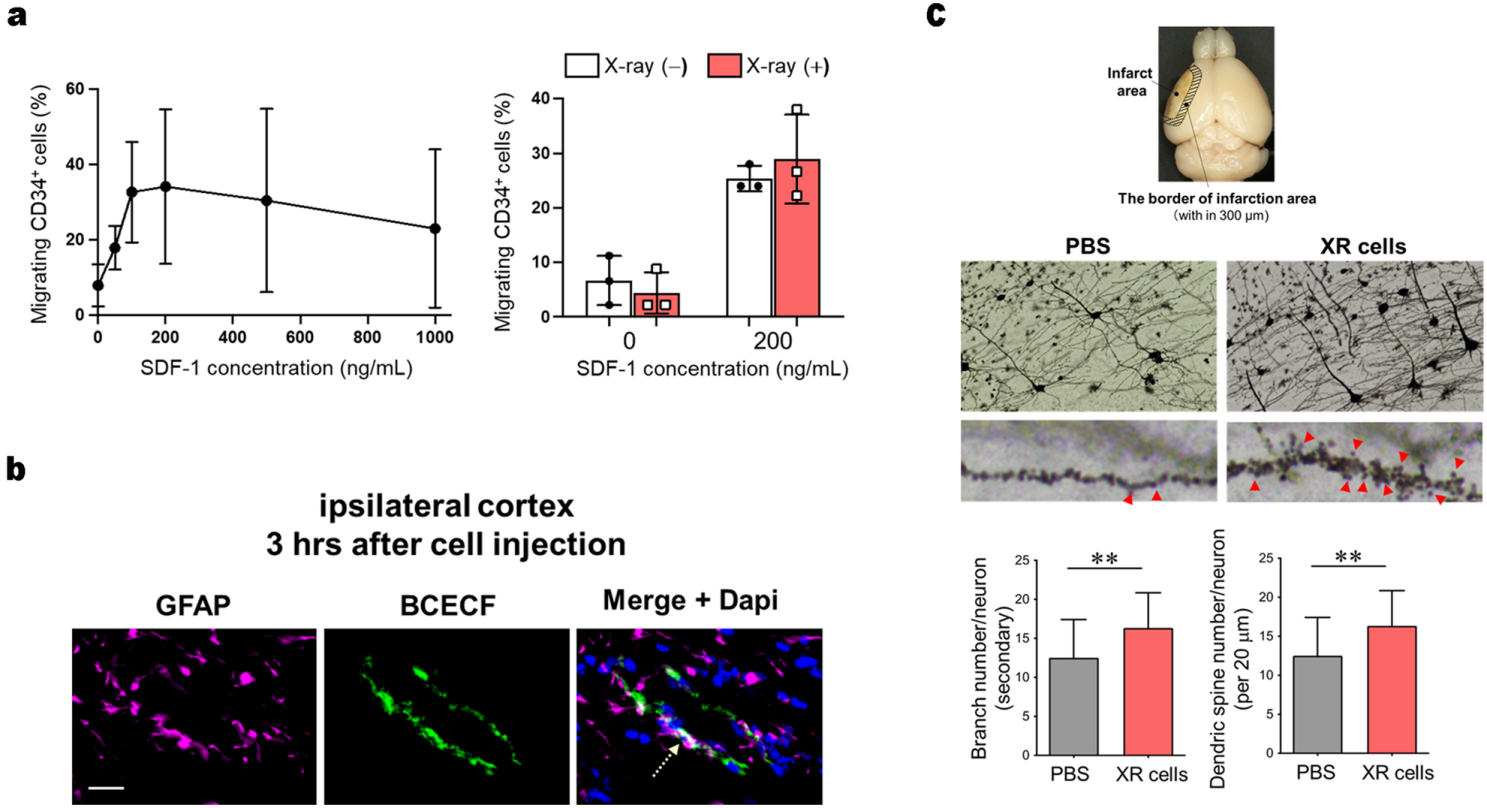
Nerve repair through direct interaction between X-irradiated mononuclear cells (XR) and astrocytes. (**a**) The optimal concentration of stromal cell-derived factor 1 (SDF-1) to maintain the migratory ability of umbilical cord blood (UCB) CD34^+^ cells was evaluated (left panel) and irradiated and non-irradiated CB UCD34^+^ cells both exhibited comparable migratory ability after SDF-1 stimulation (n = 3). (**b**) BCECF dye was transferred into astrocytes, and BCECF signals (green) were observed in glial fibrillary acidic protein (GFAP)-positive astrocytes (violet). (**c**) To measure neurite branching, 10 neurons were randomly selected from the anterior cerebral artery perfusion area within 300 µm of the border of the infarction area from each mouse, and primary and secondary branching were counted under 20 × magnification. To evaluate the spinal density, 10 neurons from each brain sample were placed in the same area where the dendritic structure was digitized using an oil immersion of 100 × magnification. The spinal density is presented as the number of spines/20 μm dendritic length (1 week; MCAO-PBS, n = 3 and MCAO-XR cell treatment, n = 3; 5 weeks; MCAO-PBS, n = 7 and MCAO-XR cell treatment, n = 9). Differences were considered statistically significant at *p* < 0.05. All results are expressed as the mean ± standard deviation (SD). The symbols ** denote *p* < 0.01.

We further investigated the precise mechanism by which the XR cells restore neuronal functions in adult mice in a short period of less than 2 months. It has been reported that CD34^+^ cells migrate towards the injured tissues in response to the stimulation by chemotactic factors such as stromal cell-derived factor-1 (SDF-1), which are released not only by the endothelium, but also by the perivascular area in the damaged tissue^11^. CD34^+^ cells derived from X-irradiated UCB exhibited the same migration ability in response to SDF-1 as their pre-irradiated counterparts (Figure 7a, Supplementary Data 1-7(a)). Importantly, fluorescent signals of the substances transferred from the XR cells were observed not only in the cerebrovascular endothelial cells but also in the astrocytes adjacent to the endothelial cells at 3 h after the administration (Figure 7b). Furthermore, the examination of synaptic conditions at the border of the infarction area revealed a significant increase in the number of branches and dendritic spines after the administration of XR cells (Figure 7c, Supplementary Data 1-7(c)). These findings strongly suggest the possibility that XR cells, in addition to their angiogenesis-promoting ability, are capable of neurogenesis-promotion by penetrating the blood-brain barrier to make direct contact with perivascular neural cells such as astrocytes.

### Adverse events after cell therapy

Mortality: In this study, 77 mice were used. The stroke model was induced in 71 mice, and all surgeries were successfully performed. Thirty-four mice survived for 37 days after model induction, and two died. One mouse in the XR cell group died 3 days after cell therapy, and one mouse in the unirradiated MNC group died 2 weeks after cell therapy. From the times of death, we considered that the deaths were likely not directly due to the administration of cell therapy.

Adverse events after cell therapy: After the behavioral evaluations were completed, pathological autopsy was performed 5 weeks after cell therapy. No abnormal findings, such as oncogenesis or inflammation, were observed visually in the lungs, liver, digestive organs, spleen, kidneys, or reproductive organs (data not shown).

## Discussion

In recent years, the clinical efficacy of cell therapy using UCB has been confirmed based on the results of clinical trials targeting various ischemic brain disorders^5,6,12^. Additionally, we previously demonstrated that UCB-derived CD34^+^ cells, but not mature cells, have a therapeutic effect on experimental stroke in mice^6^. Therefore, it is likely that CD34^+^ cells in the UCB are mainly responsible for the therapeutic effect on ischemic brain injury. However, the role of CD34^+^ UCB cells in the repair of the injured brain tissue and restoration of neurological function has not yet been fully elucidated.

In our previous study, we demonstrated an extremely interesting behavior of CD34^+^ cells, in which mouse BM MNC reached the injured brain area in the recipient mice in as little as 10 min to transfer small molecules to the vascular endothelial cells^7^. This unexpectedly rapid action of the administered cells, most likely CD34^+^ cells, led us to hypothesize that the CD34^+^ cells do not need differentiation or proliferation abilities to exert their tissue repair potential. In fact, the MNC isolated from UCB were shown to retain their tissue repair effect on experimental stroke in mice even after being X-irradiated at 15 Gy (Fig. 2e and Fig. 3). This finding raises the possibility that tissue regeneration is evoked by a completely different property of UCB cells, especially CD34^+^ cells, from conventional hematopoietic activity, in which all blood cells are made from CD34^+^ cells. Accordingly, we speculated that the therapeutic mechanism of CD34^+^ cells in cerebral infarction is as follows: there is lack of oxygen and energy sources around the infarction area due to the impaired blood flow, resulting in autophagy of cerebrovascular endothelial cells on the infarction side^7^. In contrast, CD34^+^ cells are known to be enriched in low molecular-weight energy sources such as glucose and amino acids^8^. UCB-derived MNC, once administered into the ischemic brain, immediately transfer low molecular-weight metabolites via gap junction to the vascular endothelial cells. Endogenous neural stem cells are mobilized after stroke onset, but without the presence of blood vessels at the mobilization site, the mobilized neural stem cells cannot differentiate into neurons and be engrafted. The transplanted UCB-derived MNC, specifically CD34^+^ cells, donate metabolites to the energy-deficient vessels on the injured side, preventing vessel dropout and promoting angiogenesis. The process of angiogenesis may play a crucial role in the recovery of neurological function. In addition, calcium ions, microRNA, large molecular weight proteins, and cell organelles such as mitochondria may similarly transfer through tunneling nanotubes^13,14^. Although other humoral factors such as cytokines and chemokines should also be considered, the direct cell–cell contact has been shown to be indispensable to the therapeutic effects of UCB-derived MNC, especially CD34^+^ cells, through the metabolic shift to the glycolytic system, which may in turn promote the neoneuroangiogenesis^15^.

Damaged nerve cells are regenerated by the differentiation and proliferation of neural stem cells, and the presence of blood vessels is essential for this^6^. We have previously reported that the delivery of energy sources such as glucose from hematopoietic stem/progenitor cells (HSPC) to vascular endothelial cells via gap junctions is critical for vascular regeneration in stroke^7^. We, therefore, investigated how XR cell administration improves the metabolism of the restricted site in mice using an experimental stroke model. We observed that glycolytic substrates were increased after XR cell infusion (Fig. 5a), but total adenylate levels were lower in the XR cell-treated group (Fig. 5b). There are two possible causes of low total adenylates: suppressed synthesis of adenylates or progressive degradation of adenylates, which is difficult to detect using metabolomic analysis alone. The total amount of adenylate is not constant, and there are two pathways: the salvage pathway (for breakdown and resynthesis of uric acid) and the de novo pathway. One possibility is that total adenylate levels cannot be maintained due to reduced function of these pathways. The high levels of uric acid and low levels of PRPP and IMP in the mice with experimentally induced stroke (untreated group and XR cell-treated group) may show functional reduction of both the salvage and de novo pathways. In contrast, ATP levels did not differ between model and healthy mice. It is thought that ATP is actively synthesized from ADP and AMP to maintain ATP levels. However, the ATP synthesis pathway may differ between the untreated group and the XR cell-treated group. The untreated group actively absorbs ketones from the blood to produce ATP, whereas the XR cell-treated group is thought to absorb more glucose to produce more ATP. These findings suggest that an important factor in the therapeutic effects of XR cell administration is the metabolic shift in the cerebrovascular endothelial cells around the infarction site to glycolysis, which enables the production of large amounts of ATP.

This study showed that XR cells bound directly to vascular endothelial cells and altered their metabolism to promote angiogenesis and restore blood flow, thereby regenerating damaged nerves. Further, we found that XR cells bound directly to astrocytes (Fig. 5b). Although the mechanism of interaction between XR cells and astrocytes is unknown, we predict that this may be involved in neuronal activation via Na + /K + -ATPase^16,17^. The pathway involved in the direct binding to these astrocytes may be another important pathway independent of vascular endothelial cells for neuroregeneration in the adult brain.

It was previously reported that the expression of hypoxia inducible factor-1α (HIF-1α) was enhanced in vascular endothelial cells under ischemic conditions after transplant of BM-derived MNCs^7^. A recent study claimed that newborn mice, unlike adults, can regenerate the complete heart after injury, and this regenerative capacity is lost by 7 days after birth^18^. In contrast, organisms living in hypoxic environments such as zebrafish and caudal amphibians have been shown to retain the ability to regenerate their hearts throughout their lives. Adult human hearts probably produce most of the energy through aerobic metabolism, unlike the anaerobic glycolysis system in fetal or neonatal hearts and zebrafish heart. In addition, Otsu et al.^19^ reported that hypoxia- or hypoglycemia-stimulated human peripheral blood-derived MNC showed a significant therapeutic effect on experimental stroke in rats. Taken together, it is conceivable that the neurological recovery elicited by XR cells might be attributable to a metabolic change in the glycolytic state in the damaged brain tissue induced by CD34^+^ cells, but not mature blood cells.

We must pay close attention to immune responses after UCB administration, especially regarding GVHD^20^, the most alarming allogeneic condition, as the number of cases accumulates in the future. It is considered extremely difficult to safely use immunosuppressants for GVHD prophylaxis in stroke patients, most of whom are older and exhibit a wide variety of levels of physical and metabolic function. In this study, we observed a complete interruption of the proliferative ability of T-lymphocytes (Fig. 2b) after X-irradiation at the preoptimized dose of 15 Gy, which also deprived UCB-derived CD34^+^ cells of short—(Fig. 2c) and long-term (Fig. 2d) hematopoietic activity. XR cells, thus, may be clinically applied in expectation of their neoneuroangiogenic effects without the risks of GVHD and tumorigenicity.

The microenvironment in which CD34^+^ cells reside as HSPC is called the hematopoietic niche, and is known to have a significant impact on the maintenance and exhibition of hematopoietic activity by HSPC^21^. However, there is a controversy regarding whether HSPC enter a preexisting comfortable environment or actively develop their own niches. Considering many previous reports on the ability of HSPC to engender vascular and neural tissue regeneration^22^, there is a possibility that HSPC individually but synchronically induce regeneration of vascular endothelial cells, pericytes (similar to mesenchymal stem cells^23^), and nerve cells to form their own niche^24^. In the present study, the observed effects of the XR cells support the notion that the niche-forming capacity of HSPC, at least when used for tissue repair under pathological conditions, may be independent of their hematopoietic activity. A small number of CD34^+^ cells are also present in adult PB^25–28^, although their proliferation and differentiation abilities are considerably lower than those of CD34^+^ cells in UCB, and the significance of their presence has not been clarified. Assuming that the main role of CD34^+^ cells in PB is the trigger of tissue repair, we could explain the worsening of brain, heart and other diseases using the decrease in the number of CD34^+^ cells in adult PB^28^. Similarly, CD34^+^ cells in UCB can be considered not only as the main player in neonatal hematopoiesis but also as the promoter in rapidly the expanding vascular network after birth. In conclusion, although the precise molecular mechanism underlying the tissue-repair process elicited by HSPC requires further clarification, XR cells provide an important method for elucidating the exact role of UCB in regenerative therapy.

## Materials and methods

### Preparation of XR cells

Umbilical cord blood (UCB) was collected from babies normally delivered at full-term after obtaining informed consent from all of their mothers, and all experiments using UCB were performed with the approval of the Ethics Committees of Japanese Red Cross Society, Foundation for Biomedical Research and Innovation at Kobe and Sysmex Corporation in accordance with the Declaration of Helsinki. All of the samples were processed within 72 h of collection, and the UCBs were then irradiated with X-rays, at a minimum dose of 15 Gy (MBR-1530A-TW, FUJIFILM Healthcare co., Tokyo, Japan). To evaluate the effects of the irradiation, the UCB was divided into groups, each of which was irradiated with a pre-determined dose. UCB whole blood was overlaid on Ficoll-Paque PREMIUM (Cytiva, MA, USA) density gradient media at a volume ratio of 2:1 and centrifuged at 400 g for 30 min at room temperature. After density gradient centrifugation, the mononuclear cell (MNC) layer was removed from the centrifuge tube, an equal volume of phosphate-buffered saline (PBS)(-) was added, and the suspension was centrifuged again, at 400 g for 5 min at room temperature. The supernatant was then removed from the centrifuge tube, after which the precipitate was resuspended in 10 mL PBS(-) and centrifuged again, at 400 g for 5 min at room temperature. When used without cryopreservation, the precipitates were suspended at various concentrations and used for in vitro and in vivo experiments. When used in conjunction with cryopreservation, the precipitates were suspended in a cryoprotectant solution (Stem Cell Banker GMP grade, Takara, Japan) at a density of 2 × 10^6^/mL, frozen in a programable freezer (VIA Freeze Research; Cytiva, MA, USA) at 4 °C to − 60 °C in 2 °C per minute drops, and the resulting XR cells were stored in liquid nitrogen. When used, the frozen samples were rapidly thawed in a 37 °C water bath.

### Stroke model

The animal experimentation was conducted according to the protocol and approved by the Institutional Animal Care and Use Committee of Foundation for Biomedical Research and Innovation at Kobe and Sysmex Corporation, with emphasis on accordance with ARRIVE guidelines (PLoS Bio 8(6), e1000412,2010). We used 7-week-old male severe combined immune deficiency (SCID) mice (CB-17/lcr-scid/scidJcl; Oriental Yeast, Tokyo, Japan) to create an experimental murine stroke model with excellent reproducibility, as previously described. In brief, permanent focal cerebral ischemia was induced by permanently ligating and disconnecting the distal portion of the left middle cerebral artery (MCA) using bipolar forceps under isoflurane inhalation anesthesia (3% for induction, 2% for maintenance). During surgery, the experimental animals’ rectal temperature was monitored and maintained at 37.0 ± 0.2 °C using a feedback-regulated heating pad. Cerebral blood flow (CBF) in the MCA was also monitored, and mice with a ≥ 75% decrease in CBF immediately after MCA occlusion (MCAO) were selected for use in our experiments (success rate, 100%), which were randomized and blinded.

### Cell administration

At 48 h post-MCAO, 18 experimental mice were randomly divided into 3 groups of 6 each: PBS; MNC, 1 × 10^5^ cells/mouse; and XR-MNC, 1 × 10^5^ cells/mouse. From our previous cell therapy study, as well as from a clinical perspective, 48 h post-MCAO is the optimal time for cell administration^29^. MNCs or XR-MNCs in 50 μL of PBS were administered via a 35G needle into the left common carotid artery (CCA)^29^.

### Behavioral tests

The experimental design is shown in Fig. 3a To assess motor function, post-MCAO mice were subjected to the following behavioral tests, as described below: wire-hang test; rotarod test; and open field test. All behavioral tests were performed at the optimal time, pre-determined during a preliminary study.

#### Wire-hang test

This test was performed to evaluate muscle strength or motor function. Each mouse was placed on a wire mesh plate and allowed to adapt to the environment for 5 s, after which the wire mesh plate was gently inverted and secured to the top of an open-top glass box (25 × 25 × 25 cm). Latency to fall was measured, using a maximum trial duration of 3 min, repeating the trial 5 times at 1 min intervals.

#### Rotarod test

This test was performed to evaluate sensorimotor skills. Each mouse was placed on a stationary rotarod drum (Muromachi Kikai Co., Ltd., Tokyo, Japan) for 5 s, after which the rotor was turned on. The rotarod drum was then accelerated from 4 to 40 rpm over a period of 5 min, and the time it took for each mouse to fall off the rotating drum was recorded. The experiment was repeated 5 times at an interval of 1 min, and the average time for each of the five falls was used for statistical analysis.

#### Open field test

This test was performed to evaluate spontaneous activity. The experimental mice were place into a 25 × 25 × 25 cm box, after which they were allowed to roam freely for 30 min in a light environment, followed by 30 min in a dark environment (Taiyo Electric Co., Ltd., Osaka, Japan). Infrared beams were mounted horizontally and vertically at specified intervals on the X-, Y-, and Z-banks of the box, and the total number of beam crossings was counted for each animal, and scored as locomotion for horizontal movement.

### Sample collection for metabolomic analysis

Brain samples were collected for metabolomic analysis 24 h after cell therapy. The mice were euthanized and then decapitated, and the whole heads were immediately immersed in liquid nitrogen. After removing the skull, the brains were dissected on dry ice into two sections, ipsilateral and contralateral to the infarct area. All tissue specimens weighed 35.5–40.4 mg and were stored at − 80 °C until measured (n = 5).

### Metabolomic analysis

The experimental specimens were analyzed for 900 metabolites via capillary electrophoresis time-of-flight mass spectrometry (CE-TOF MS), in order to evaluate the influence of the MCAO and the effects of the subsequent cell therapies.

These analyses were performed by Human Metabolome Technologies (Tsuruoka, Japan). Each frozen sample was homogenized in 50% acetonitrile containing 10 µM internal standard, and the samples were homogenized twice using a tissue homogenizer. After centrifugation (2 cycles at 2300 G, 4 °C, 5 min), 400 µL of the supernatant was transferred to an ultrafiltration tube (Ultra-free MC PLHCC; Human Metabolome Technologies, Tsuruoka, Japan), centrifuged again (9100 G, 4 °C, 120 min), and then subjected to ultrafiltration. The filtrate was dried, and samples < 5 mg were redissolved in 25 μL of Milli-Q, while samples > 5 mg were redissolved in 50 μL of Milli-Q. The redissolved samples were used for CE-TOF MS, performed using an Agilent CE-TOF MS system (Agilent Technologies, CA, USA).

Cationic metabolites were analyzed using a fused silica capillary (i.d. 50 μm × 80 cm) with cation buffer solution (Human Metabolome Technologies, Tsuruoka, Japan) as the electrolyte. The samples were injected at a pressure of 5.0 kPa for 10 s, and 30 kV CE voltage was applied. Electrospray ionization mass spectrometry (ESI–MS) was performed in the positive ion mode at a capillary voltage of 4000 V, and the specimen was scanned with the spectrometer at a mass-to-charge ratio (m/z) of 50–1000. The other conditions were the same as those used for the cation analysis.

Anionic metabolites were analyzed using a fused silica capillary (i.d. 50 μm × 80 cm) with an anion buffer solution (Human Metabolome Technologies, Tsuruoka, Japan) as the electrolyte. Samples were injected at a pressure of 5.0 kPa for 25 s (∼25 nL), and 30 kV CE voltage was applied. ESI–MS was performed in the negative ion mode, at a capillary voltage set at 3500 V. The sample was then scanned using a spectrometer from m/z 50 to 1000. The metabolites found in the samples were identified by comparing their migration time and m/z ratio with those of authentic standards, and were quantified by comparing their peak areas with those of authentic standards using Chem Station software (Agilent Technologies). Metabolomic data were processed via principal component analysis (PCA) and hierarchical cluster analysis (HCA) using software (Human Metabolome Technologies, Tsuruoka, Japan). All groups, n = 5.

### Low-molecular-weight fluorescence markers in cytoplasm of XR cells

XR cells were incubated with 5 µmol/L 2′,7′-bis-(2-carboxyethyl)-5-(and-6)-carboxyfluorescein acetoxymethyl ester (BCECF-AM; Dojindo, Kumamoto, Japan) for 30 min at 37 °C. BCECF-AM was then converted to BCECF in the cytoplasm, and BCECF-loaded XR cells were washed twice with PBS prior to being used for in vivo experiments. The cells were diluted to the appropriate concentration with PBS, and used immediately for experiments. At 48 h post-MCAO, 1 × 10^5^ BCECF-loaded XR cells in 50 µl PBS/mouse were injected into the carotid artery. At 10 min, 30 min, 1 h, and 3 h post-cell transplantation, the mice were euthanized and then decapitated (n = 3). After removal from the skull, the brains were sectioned coronally (20 µm) using a vibratome (Leica, Wetzlar, Germany), and stained with antibodies against CD31 (BD Biosciences, NJ USA) and glial fibrillary acidic protein (GFAP; Thermo Fisher Scientific, MA, USA). Primary antibodies were visualized using Alexa Fluor 555- or 647-conjugated secondary antibodies (Molecular Probes, OR, USA), and the cell nuclei were stained with 4′,6-diamino-2-phenylindole (DAPI; Kirkegaard & Perry Laboratories, MD, USA).

### Cerebral blood flowmetry

CBF was measured via laser speckle flowmetry (OmegazoneOZ-1; Omegawave Inc., Tokyo, Japan), using an intact skull after a scalp incision under isoflurane anesthesia. The infarct area in the ipsilateral hemisphere, along with its corresponding region in the contralateral hemisphere, were defined as regions of interest (ROIs). Five consecutive raw speckle images were acquired every second and averaged, with data presented as the ratio of ipsilateral/contralateral CBF, as follows: 1 week (no surgery, n = 3; MCAO-PBS, n = 3; MCAO cell treatment, n = 3) and 5 weeks (MCAO-PBS, n = 7; MCAO cell treatment, n = 9).

### Golgi staining and dendrite spine measurement

Golgi-Cox staining was performed using a SuperGolgi kit (Bioenno Lifesciences, CA, USA) according to the manufacturer’s instructions. In brief, the experimental mice were perfused with saline for 5 min, after which the brains were immersed in solution A for 10 days. The brains were then rinsed with distilled water and transferred to solution B for 2 days at room temperature. The brains were sectioned into coronal slices (100 µm) using a vibratome (Leica, Wetzlar, Germany), and staining and washing were performed according to the manufacturer’s protocols. To measure neurite branching, 10 neurons per mouse were randomly selected from within 300 µm of the infarct border of the anterior cerebral artery perfusion area, and the primary and secondary branches were counted under 20 × magnification using a BZ-X800L fluorescence microscope (KEYENCE, Osaka, Japan). To evaluate dendrite spine density, 10 neurons from each brain sample, in the same region as described above, were counted under a 100 × oil immersion objective, with the density expressed as the number of spines/20 μm dendrite length.

### Microangiography and image analysis

Microangiographic images of the mouse brain were obtained using monochromatic synchrotron radiation (SR) at the Japan Synchrotron Radiation Research Institute (SPring-8, Hyogo, Japan), which is one of three large third-generation synchrotron radiation facilities worldwide (the other two facilities are the Advanced Photon Source at Argonne National Laboratory in the United States and the European Synchrotron Radiation Facility in Grenoble, France). These facilities are open to scientists from a variety of fields, including material, chemical, and life sciences. We have previously described the experimental setup for X-ray imaging using monochromatic SR at the SPring-8 BL28B2 beamline in a previous study. The storage ring was operated at an electron beam energy of 8 GeV and a beam current of 80–100 mA, with a distance between the point source in the bending magnet and the detector of approximately 45 m. A nearly parallel X-ray beam was used for imaging without blurring due to beam divergence because of the small size of the X-ray source and extremely long source-to-object distance. The single crystal monochromator utilized a single energy source for SR, and the shutter system was located between the monochromator and the object. The X-rays transmitted through the object were detected by a direct-conversion detector incorporating a saticon pickup tube. The monochromatic X-ray energy was adjusted to 37.5 keV, just above the K-edge energy for barium, to produce the highest-contrast image of the barium. In the imaging experiments, the X-ray flux at the position of the object was approximately 1 × 10^10^ photons/mm^2^/s, and the images were acquired at 1024 × 1024 pixels with a 10-bit resolution after analog-to-digital conversion. The field of view (FOV) was 4.5 × 4.5 mm^2^, and pixel size was  ∼ 4.5 μm.

### CD34^+^ cell purification by immunomagnetic cell sorting

For CD34^+^ cell purification, UCB-derived MNC were subjected to immunomagnetic separation using a human CD34 Microbead kit (Miltenyi Biotech, Bergisch Gladbach, Germany), according to the manufacturer’s instructions. In brief, UCB-derived MNC were resuspended in PBS supplemented with 0.5% bovine serum albuim (BSA) and 5 mM ethylenediaminetetraacetic acid (EDTA; PBS-BSA-EDTA) at a concentration of 1 × 10^8^ cells per 300 μL, and incubated with 100 μL FcR blocking reagent and 100 μL CD34 microbeads for 30 min at 6 °C. After washing with PBS-BSA-EDTA, the labelled cells were filtered through a 30 μm nylon mesh (Miltenyi Biotech, Bergisch Gladbach, Germany) and loaded onto a column (Miltenyi Biotech) installed in a magnetic field. The captured cells were eluted after the column was removed from the magnet, and the collected cells were loaded onto a second column, after which the purification step was repeated. The purity of the isolated CD34 + cells was generally > 95%, as assessed by flow cytometry (FCM) using FACSCanto II (Becton Dickinson, CA, USA).

### T lymphocyte purification by immunomagnetic cell sorting

For T lymphocyte isolation, UCB-derived MNC were subjected to immunomagnetic separation using a Human Pan T cell Isolation kit (Miltenyi Biotech) according to the manufacturer’s instructions. In brief, UCB-derived MNC were resuspended in PBS at a concentration of 1 × 10^7^ cells per 40 μL and incubated for 5 min in a refrigerator (2–8 °C) with 10 μL of Pan T cell biotin-antibody cocktail containing biotin-conjugated monoclonal antibodies against CD14, CD15, CD16, CD19, CD34, CD36, CD56, CD123, and CD235a (glycophorin-A). After adding 30 μL of PBS-BSA-EDTA, UCB-derived MNCs were incubated for an additional 10 min in a refrigerator with 10 μL of microbeads conjugated with an anti-biotin monoclonal antibody. After washing with PBS-BSA-EDTA, the labelled cells were filtered through a 30 μm nylon mesh and loaded onto a column placed in a magnetic field. The flow-through cells were collected and reapplied to the second column, as described above. The purity of the isolated CD3^+^ T lymphocytes was generally > 95%, as assessed by FCM.

### Colony forming unit (CFU) assay

Purified CD34^+^ cells, whether irradiated or not, were suspended in Iscove’s modified Dulbecco’s medium (IMDM; Thermo Fisher, MA, USA) containing 2% fetal bovine serum (FBS; Thermo Fisher Scientific) at a concentration of 1 × 10^3^ to 1 × 10^5^ cells/mL. The cell suspensions were mixed with 10 volumes of methylcellulose-based semi-solid culture medium (MethoCult H4034 Optimum; StemCell Technologies, Vancouver, Canada). Aliquots of the mixture, containing the specified number of CD34^+^ cells per 1.1 mL, were plated in duplicate in 35 mm dishes and incubated for 14 days at 37 °C in a humidified atmosphere containing 5% CO_2_. Each plate was scored for burst forming unit erythroid (BFU-E), granulocyte–macrophage colony-forming unit (CFU-GM), and mixed lineage colony-forming unit (CFU-mix) colonies.

### T lymphocyte activation assay

Isolated T lymphocytes, whether irradiated or not, were resuspended in PBS at a concentration of 1 × 10^7^ cells per 40 μL. The T lymphocytes were then cultured with anti-CD3/CD28 monoclonal antibody (mAb)-coated Dynabeads (Dynabeads Human T-Activator CD3/CD28, Thermo Fisher) at a concentration of 1 × 10^7^ cells per 5 μL. After the addition of interleukin (IL)-2 (Chiron, Emeryville, CA, USA) on day 0 to obtain a final concentration of 100 g/mL, the cells were subcultured every 3 days during the rapid growth phase in Roswell Park Memorial Institute (RPMI) 1640 (Thermo Fisher Scientific) supplemented with 10% fetal calf serum (FCS; Thermo Fisher Scientific), L-glutamine (Thermo Fisher), and penicillin plus streptomycin (Thermo Fisher). The CD3 antigen expression of the cultured T lymphocytes was assessed by FCM using an allophycocyanin (APC)-labelled anti-CD3 antibody (Becton Dickinson). The kinetics of apoptosis in the cultured T lymphocytes were evaluated two-color immunolabelling with fluorescein isothiocyanate (FITC)-labelled annexin-V (Nacalai Tesque, Kyoto, Japan) and 7AAD (Becton Dickinson).

### Uptake of human vascular endothelial growth factor (VEGF) by human umbilical vein endothelial cells (HUVECs)

HUVECs (Kurabo, Osaka, Japan) were cultured in HuMedia-EB2 (Kurabo, Osaka, Japan) according to the manufacturer’s protocol, and passage 6 HUVECs were used for all experiments. Recombinant human VEGF (rhVEGF; R&D Systems, Minneapolis, MN, USA) was assessed using a method described previously. In brief, biotin-conjugated rhVEGF was incubated with streptavidin-conjugated APC (Becton Biosciences) at a molecular ratio of 4:1 for 10 min at room temperature. HUVECs were harvested and suspended in PBS containing 1% FBS at a density of 1 × 10^5^ cells/mL. A total of 1 × 10^6^ UCB-derived MNCs, or a specified number of the isolated CD34^+^ cells (1 × 10^2^ to 1 × 10^5^ cells) and APC-labelled rhVEGF (at a final concentration of 10 nM), were simultaneously added to 1 × 10^5^ HUVEC and incubated at 37 °C for 3 h. HUVEC were incubated with MNC or CD34^+^ cells separated in a semi-permeable membrane chamber (Costar, TN, USA) to evaluate the effect of humoral factors on VEGF uptake by HUVEC. The mixed cell suspension was washed twice with PBS and stained with a phycoerythrin (PE)-conjugated anti-human CD31 antibody (Becton Dickinson), FITC-conjugated anti-human CD45 antibody (Becton Dickinson), and 7-AAD. The intensity of APC fluorescence in CD31^+^CD45^-^7AAD HUVEC was assessed using FCM.

### Migration assay

Migration assays were performed in 6.5 mm diameter trans-well plates (Costar) with 5 μm pore filters as previously described^30^. The upper and lower compartments of the transwell were separated using a filter coated overnight at 4 °C with fibronectin (Sigma, MO, USA) at a concentration of 20 g/mL in PBS. Before the cells were added to the upper compartment, the coated transwells were washed three times with the assay medium (IMDM containing 0.25% BSA). Freshly isolated CD34^+^ cells (1 × 10^5^ cells), regardless of exposure to a 15 Gy dose of X-rays, suspended in 0.1 mL of assay medium, were added to the upper compartment, while 0.6 mL of assay medium in the presence or absence of rhSDF-1 (200 ng/mL, PeproTech, NJ, USA) was added to the lower compartment. Preliminary experiments showed that the optimal concentration of SDF-1 to induce transwell migration of UCB-derived CD34^+^ cells was 200 ng/mL. Transwell plates were incubated at 37 °C with 5% CO_2_ for 4 h. Cells that migrated to the bottom compartment were counted under a microscope.

### Targeted metabolite detection in MNCs

The CellTiter-Glo® Luminescent Cell Viability Assay (G9241 Promega, WI, USA) was used to quantify a standard curve of different concentration of an ATP solution. Add a volume of CellTiter-Glo® 2.0 Reagent equal to the volume of medium containing cells. The contents were then mixed for 2 min on an orbital shaker to induce cell lysis. The plate was incubated at room temperature for 10 min to stabilize the luminescent signal, and the luminescence was recorded. The levels of glucose, glutamate, and lactate were determined using a glucose, glutamate, or lactate Glo assay kit (J6021, J5021, and J7021, Promega). Both X-ray (-) and X-ray ( +) cells were washed in cold PBS, immediately inactivated with 0.6 N HCl to halt metabolic activity, and neutralized with 1 M Tris base. The resulting lysate was combined at a 1:1 ratio with each detection reagent and luminescence was measured using a microplate reader (GloMax® Discover System, GM3000, Promega).

### SCID-repopulating cell (SRC) assay

Animal experiments were approved by the Animal Care Committee of the Japanese Red Cross Society and performed in accordance with ARRIVE guidelines. 5-week-old NOD.Cg-PrkdcscidIl2rgtm1Sug/ShiJic (NOG/SCID) 14 mice were obtained from Central Institute for Laboratory Animals (Kawasaki, Japan). All the mice were handled under sterile conditions and maintained in aseptic isolators at the Laboratory Animal Facility of CLEA Japan, Inc. In this study, purified 1 × 10^6^ UCB-derived irradiated CD34^+^ cells as test samples or 5 × 10^4^ UCB-derived unirradiated CD34^+^ cells as positive controls were infused via the tail vein into mice exposed to a sublethal dose of X-rays (2 Gy). The mice were sacrificed 12 weeks after CD34^+^ cell infusion, and the peripheral blood of each mouse was washed in α Minimum Essential Medium (MEM) containing 10% FCS. The percentage of human CD45^+^ cells in the mouse peripheral blood was analyzed using FCM. Mice were considered positive if > 1% of the total mouse bone marrow cells were human CD45^+^.

### Statistics

Statistical analyses in Figs. 1 and 2 were performed using GraphPad Prism 8 (GraphPad Software, San Diego, CA, USA). The results of metabolites are expressed as mean ± standard error of the mean (SEM) and were analyzed using paired t-tests. Results with *p*-values < 0.05 were considered statistically significant.

 Statistical analyses in Figs. 3, 4, 5,6 and 7 and Supplementary Figure S1 were performed in GraphPad Prism 9 (GraphPad Software). Results of in vitro tests, behavioral tests, cerebral blood flow ratios, and immunohistological staining are expressed as mean ± standard deviation (SD). In all tests, differences between groups were analyzed using the Bonferroni test for correction for multiple comparisons. Because these results were not normally distributed, they were assessed using the Kruskal–Wallis test followed by Dunn’s multiple comparisons test. The results of metabolites are expressed as mean ± standard error of the mean (SEM) and were analyzed using one-way ANOVA with Dunn’s test. Results with *p*-values < 0.05 were considered statistically significant.

### Supplementary Information

Supplementary Figure S1.

Supplementary Information 2.

Supplementary Information 3.

Supplementary Information 4.

Supplementary Information 5.

Supplementary Legends.

## Data Availability

All data generated or analysed during this study are included in this published article and its supplementary information files.
