# Supplementary figures and images for: X-irradiated umbilical cord blood cells retain their regenerative effect in experimental stroke
Source: Sci Rep. 2024 Mar 22;14:6907. doi: 10.1038/s41598-024-57328-z (PMC10959937; doi:10.1038/s41598-024-57328-z)

## Slide 1
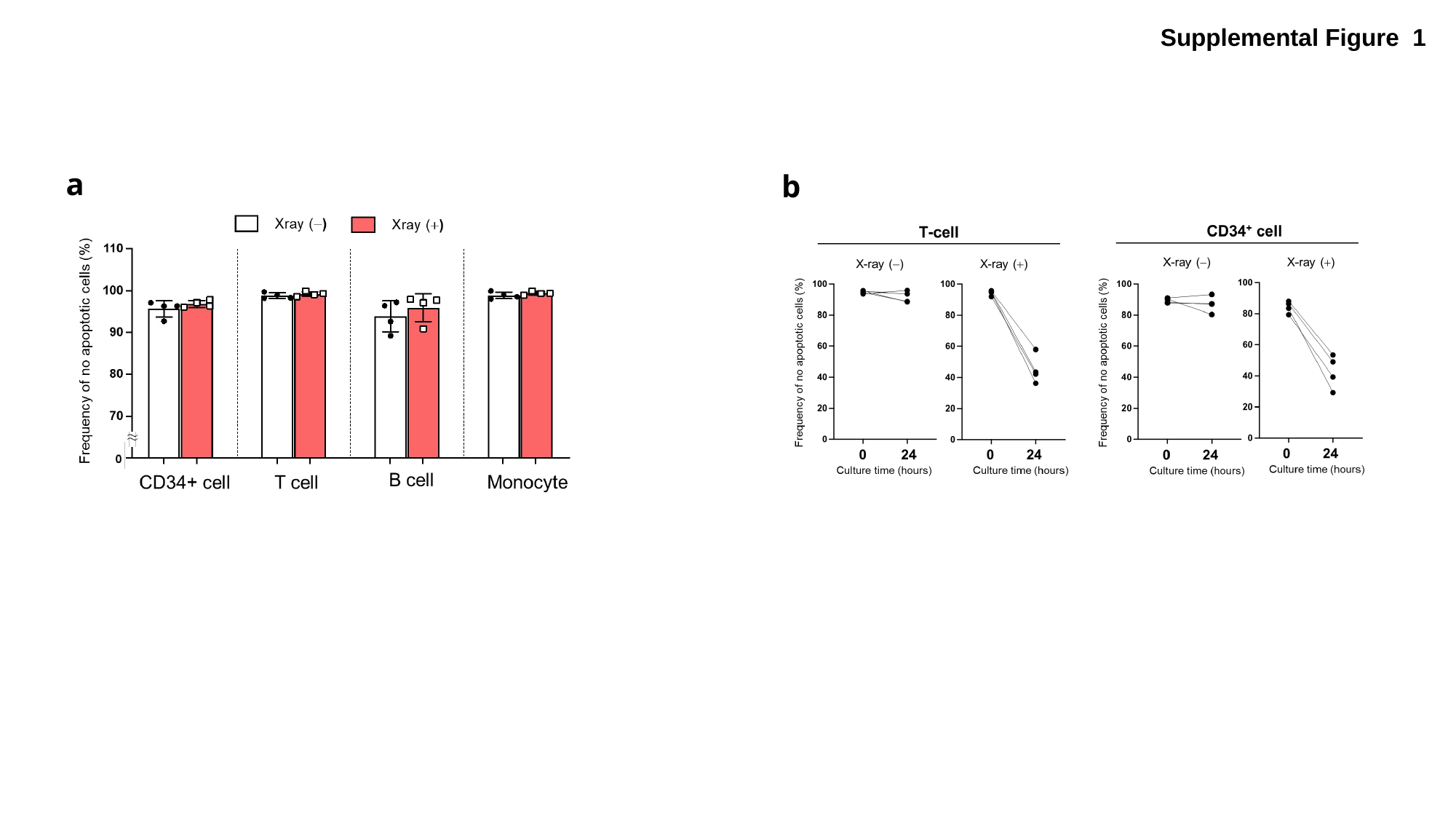

Supplemental Figure 1
a
b

Supplement: Supplementary file 1 — Supplementary Figure S1. [file 41598_2024_57328_MOESM1_ESM.pptx]
