# Supplementary material for: X-irradiated umbilical cord blood cells retain their regenerative effect in experimental stroke
Source: Sci Rep. 2024 Mar 22;14:6907. doi: 10.1038/s41598-024-57328-z (PMC10959937; doi:10.1038/s41598-024-57328-z)
Supplement: Supplementary file 6 — Supplementary Legends. [file 41598_2024_57328_MOESM6_ESM.docx]

**Supplementary Figure S1: Effect of X-irradiation on immunophenotype and cellular composition**

(a) No cells exhibiting apoptotic features were observed in any type of UCB-derived MNC, including CD34^+^ HSPC, T lymphocytes, B lymphocytes, and monocytes, for at least 48 h after irradiation (n = 5), as determined by the upregulation of cell surface phosphatidylserine and 7-amino-actinomycin D penetration. (b) Irradiated T lymphocytes and CD34^+^ cells, stimulated by growth signals, turned around and underwent apoptosis within 24 hours, while apoptotic features were not observed in the unirradiated T lymphocytes or CD34^+^ cells. All results are expressed as mean ± standard deviation (SD).
